# Seroprevalence and Serotypes of Dengue Virus Infection in Ghana: A Systematic Review and Meta-Analysis

**DOI:** 10.3390/diseases13040114

**Published:** 2025-04-14

**Authors:** Isaac Boamah, Alex Odoom, Kwamena W. C. Sagoe, Eric S. Donkor

**Affiliations:** Department of Medical Microbiology, University of Ghana Medical School, Korle Bu, Accra P.O. Box KB 4236, Ghana; ikebosa@yahoo.com (I.B.); alexodoom2018@gmail.com (A.O.); ksagoe@ug.edu.gh (K.W.C.S.)

**Keywords:** dengue virus, seroprevalence, serotypes, Ghana, vector control, systematic review

## Abstract

Background: Dengue virus (DENV) infection poses a serious and growing public health threat in many tropical and subtropical regions worldwide, including Ghana. Despite the heightened risks due to suitable climatic conditions and the presence of competent vectors, the epidemiology of DENV in Ghana remains poorly understood, underscoring the critical need for a comprehensive assessment of the burden and circulation of the virus in the country. Methods: The review was conducted in accordance with the PRISMA guidelines. A literature search was conducted using the PubMed, Web of Science, and Scopus databases from inception to 24 September 2024. Studies presenting primary DENV seroprevalence data among Ghanaian populations were included. Quality was assessed using JBI tools. Meta-analyses estimated pooled prevalence with 95% CIs using random-effects models. Heterogeneity was evaluated using I^2^ statistic. Results: A total of nine studies met the selection criteria, with eight studies involving febrile patients and one involving blood donors. The pooled seroprevalence rates were 33.3% (95% CI: 16.2–50.4%) for IgG, 5.9% (95% CI: 0–12.5%) for IgM, 1.5% (95% CI: 0.4–2.6%) for DENV RNA, and 32.3% (95% CI: 0–68.5%) for IgG/IgM combined. Notably, the IgG seroprevalence exhibited substantial heterogeneity (I^2^ = 99%). DENV-2 and DENV-3 were the predominant serotypes identified. There was potential bias in seroprevalence estimates from hospital-based febrile samples. Conclusions: This review has established the prevalence and circulation of DENV serogroups in Ghana. The higher seropositivity and heterogeneity underscore the need for standardized large-scale surveillance to optimize disease characterization and guide control. Integrating dengue prevention into existing vector control programs could help reduce risks in Ghana.

## 1. Introduction

Dengue virus (DENV) is one of the fastest-spreading mosquito-borne viruses worldwide [1]. DENV belongs to the Flaviviridae family and is an RNA virus with a single-stranded, positive-sense genome of approximately 10.7 kilobases. The viral genome encodes a single polyprotein that is processed into three structural proteins (Capsid, Membrane, and Envelope) and seven non-structural proteins (NS1, NS2A, NS2B, NS3, NS4A, NS4B, and NS5) [2]. The spherical DENV virion has a diameter of around 50 nanometers, with the envelope (E) protein playing a crucial role in host cell attachment and membrane fusion, initiating the infection process [3]. The viral non-structural proteins have diverse functions crucial for DENV replication and pathogenesis. The NS1 protein is involved in RNA replication and immune system modulation, while the NS3 protein serves as a protease, helicase, and RNA triphosphatase, enabling viral genome replication. The NS5 protein contains RNA-dependent RNA polymerase and methyltransferase activities, essential for viral RNA synthesis and modification [4]. Understanding the role of these proteins, particularly NS1, in immune evasion and triggering a cytokine storm is critical for explaining the severity of dengue disease and patient morbidity. The complex interplay between DENV proteins and the host’s immune system contributes significantly to the virulence of this important arboviral pathogen [5].

DENV infection is caused by any of the four DENV serotypes (DENV-1 to DENV-4) and presents a significant public health challenge in tropical and subtropical areas [6]. Clinically, DENV infection varies from asymptomatic cases to mild fever, and in severe cases, it can become life-threatening. Early symptoms include high fever, severe headaches, pain behind the eyes, muscle and joint pain, nausea, vomiting, skin rash, and minor bleeding [7]. In more severe cases, the disease can progress to a critical phase with increased capillary permeability and plasma leakage, leading to hypovolemic shock [8]. Without timely treatment, complications such as organ failure, severe bleeding, and circulatory collapse can occur, often resulting in death [9]. Dengue remains a leading cause of hospitalization and death in both children and adults in endemic regions [10].

DENV is transmitted primarily by Stegomyia (formerly *Aedes*) *aegypti* and Stegomyia *albopictus* mosquitoes [11]. DENV transmission is expanding due to rising global temperatures and increased urbanization, leading to significant health, social, and economic impacts [12]. The World Health Organization (WHO) estimates that there are approximately 390 million infections annually, with over 3.9 billion people in more than 100 countries at risk [7]. By the end of April 2024, the WHO had recorded approximately 7.6 million cases of dengue fever thus far in 2024, with over 3.4 million clinically confirmed infections, 16,000 severe cases, and fatalities surpassing 3000 [13].

Latin American and Asian countries are experiencing hyperendemic transmission with frequent outbreaks [14,15]. In Africa, dengue has historically been underreported compared with other regions, but in the past two decades, cases have sharply increased, particularly in West and Central Africa, indicating that the true burden of the disease may be underestimated [16]. In Ghana, sporadic dengue cases have been reported since the 1960s, with the first major outbreak documented in 2016 affecting over 1061 people [17].

While Ghana is on the fringes of the WHO’s DENV endemic map, some regional hospital-based studies provide serological evidence of previous exposure in healthy individuals as well as acute febrile patients [18,19]. However, the nationwide seroprevalence is undefined, and the full spectrum of the disease remains unclear because of the absence of systematic entomological and virological surveillance. The lack of detailed characterization poses challenges for effective prevention. This underscores the rationale for conducting an extensive review of all available evidence. This review aims to provide a comprehensive synthesis of the pooled prevalence of DENV infection markers—including DENV-specific IgG, IgM, and DENV RNA, as well as circulating serotypes—in Ghana to facilitate well-informed public health response planning.

## 2. Materials and Methods

This review was conducted following the Preferred Reporting Items for Systematic Reviews and Meta-Analyses (PRISMA) guidelines [20] to ensure a rigorous and transparent methodology. It should be noted that the review was not registered in a public registry prior to its conduct.

### 2.1. Information Sources and Search Strategy

Three databases (PubMed, Web of Science, and Scopus) were searched for relevant publications up to 24 September 2024. The following search terms were used in various combinations: (“Dengue virus” OR “DENV” OR “Dengue fever”) AND (“Ghana”) AND (“Epidemiology” OR “Seroprevalence” OR “Diagnosis” OR “Surveillance” OR “Case report” OR “Outbreak”). Additional studies were identified through manual searches of references from eligible articles and relevant reviews on dengue in Africa. No restrictions were placed on the study design or sample size. The relevant articles were imported into Mendeley Reference Manager Version 2.122.1 for processing and organization.

### 2.2. Study Selection and Data Extraction

All records obtained from searches were imported into Rayyan software [21]. Two reviewers independently screened the records according to predefined eligibility criteria. Specifically, the relevance of each study for investigating DENV epidemiology in Ghana through the detection of biological markers was assessed. Potentially eligible full texts were retrieved and evaluated. A standardized data extraction form was developed in Microsoft Excel and pilot-tested on three randomly selected studies before being finalized. The following characteristics were extracted from the included studies by one reviewer and verified by another:

1. Author and year of publication;
2. Study design (e.g., cross-sectional, case–control);
3. Study setting and period;
4. Time frame (e.g., outbreak, non-outbreak);
5. Target population and inclusion/exclusion criteria;
6. Sample size;
7. Types of biological samples (e.g., blood, serum, plasma);
8. Dengue diagnostic tests used (e.g., IgM ELISA, IgG ELISA, RT–PCR);
9. Main outcome variables (e.g., IgG seroprevalence, IgM seroprevalence, and DENV RNA detection);
10. DENV serotypes identified.

Any discrepancies in the extracted data between reviewers were resolved by re-examining the original text. Incomplete or unreported information was documented accordingly. The extracted data were compiled into evidence tables for analysis and visualization of key parameters across studies. This standardised methodology ensured accurate extraction of relevant details to address the review objectives.

### 2.3. Inclusion Criteria

The following inclusion criteria were applied during the study selection process:

1. Primary research studies conducted among humans of all age groups in Ghana;
2. Studies employing serological and/or molecular techniques to detect DENV immunoglobulins (IgG and IgM) and/or RNA;
3. No date restrictions were placed on the included studies;
4. Studies providing key details on methodology, study characteristics (design, location, population), diagnostic methods used and main findings;
5. Studies from all geographical regions and population groups across Ghana.

### 2.4. Exclusion Criteria

Studies were excluded if they met any of the following criteria:

1. Review articles, systematic reviews, meta-analyses, case reports, opinion pieces, letters, and conference abstracts—as these do not report primary research;
2. Studies conducted outside Ghana or not among human populations;
3. Studies detecting antibodies or antigens of pathogens other than dengue virus;
4. Studies focusing only on vector surveillance or reporting vector indices without dengue virus detection;
5. Studies with incomplete/insufficient methodological information to assess quality and extract relevant data;
6. Studies published in languages other than English.

These clear inclusion and exclusion criteria helped obtain a focused set of studies relevant to the objectives of the review.

### 2.5. Quality Assessment

Study quality was assessed using the Joanna Briggs Institute (JBI) Critical Appraisal tool relevant for cohort, cross-sectional, and case–control study designs [22]. A customized nine-item checklist was used to examine methodological domains:

1. Was the sample frame appropriate to address the target population?
2. Were study participants sampled appropriately?
3. Was the sample size adequate?
4. Were the study subjects and setting described in detail?
5. Was data analysis sufficiently comprehensive of the identified sample?
6. Were valid methods used for condition identification?
7. Was the condition measured reliably for all participants?
8. Was statistical analysis appropriate?
9. Was the response rate adequate, and if not, was this managed appropriately?

Each item was scored “1” if adequately met and “0” if unreported or poorly described. The maximum quality score was 9. Studies scoring ≥ 7 points were considered “high” quality, those scoring 4–6 points were considered “moderate”, and those scoring <4 points were considered “low”. The quality assessment was independently conducted by two reviewers, with a third reviewer resolving any discrepancies.

### 2.6. Statistical Analysis

The meta-analyses were performed using R software version 4.2.1 and applicable R packages. Specifically, the metafor package was used to conduct all the analyses. Random effects meta-analyses generated pooled proportions with 95% confidence intervals (CIs) for DENV IgM, IgG, and RNA detection and combined IgG/IgM results. Heterogeneity was assessed via the I^2^ statistic, with values greater than 50% indicating moderate to high heterogeneity. Forest plots graphically display prevalence outcomes with study-specific CIs and pooled estimates. Publication bias was evaluated using funnel plots.

## 3. Results

### 3.1. Search Results

The database searches (PubMed, Scopus, and Web of Science) yielded 152 publications. An additional 10 studies were identified through manual techniques and reference screening, totaling 162. After 90 duplicate reports were removed, 72 studies underwent title/abstract review. Most (*n* = 53) did not meet the criteria and were excluded at this stage. The remaining 19 papers underwent full-text assessment; 10 were excluded for the following reasons:

1. Study setting not specified (*n* = 1);
2. Other pathogens/vectors studied (*n* = 7);
3. Unable to access the full text (*n* = 2).

**Figure 1:**
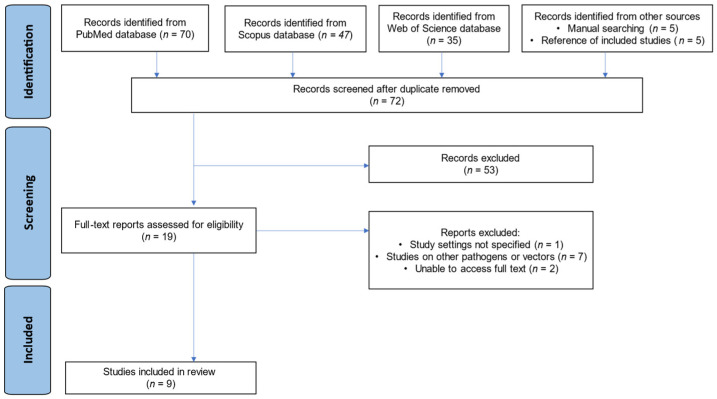
PRISMA flow diagram showing the study selection process.

Finally, 9 studies [18,19,23,24,25,26,27,28,29] met all eligibility criteria and were included in the review. Any discrepancies in study selection between reviewers were resolved via discussion until consensus was reached. The overall selection process is summarized in a PRISMA flow diagram (Figure 1).

### 3.2. Study Characteristics

**Figure 2:**
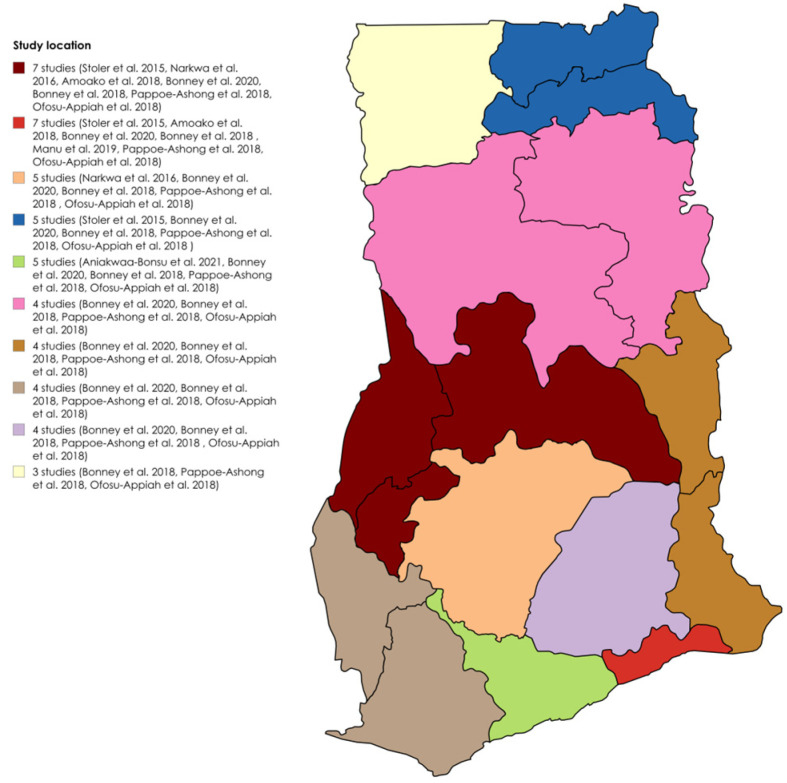
Geographical distribution of DENV seroprevalence studies in Ghana [18,19,23,24,25,26,27,28,29].

The detailed study characteristics are provided in Table S1. Six studies utilized a cross-sectional design [19,23,24,27,28,29], one was a case study [25], and two did not specify the design [18,26]. Two identified infections during outbreaks [26,28], whereas seven detected infections outside of outbreaks [18,19,23,24,25,27,29]. All were clinical-based. Five studies used both RT–PCR and ELISA [18,19,26,27,29], one used both RDT and ELISA [23], two used only RT–PCR [24,25], and one used ELISA alone [28]. Seven studies detected IgG [18,19,23,26,27,28,29], five detected IgM [18,23,26,27,29], three detected RNA [24,25,26], and three detected IgG/IgM [23,26,27]. Three studies identified DENV-2 [24,25,26], and one identified DENV-3 [26]. The target populations included patients aged 6 months to 82 years with suspected yellow fever [29]; children aged 2–14 years with confirmed malaria [18]; patients with fever and jaundice [28]; healthy blood donors [19]; suspected dengue- and/or chikungunya-fever patients [27]; patients suspected of Ebola virus disease [26]; suspected viral hemorrhagic fever cases [25]; children aged 1–15 years presenting with fever [24]; and adults aged ≥18 years with fever and three malaria-like symptoms [23]. The most dominant sampled regions for DENV seroprevalence studies in Ghana were Greater Accra and Brong Ahafo, both included in seven out of the nine studies, followed by Ashanti in five out of nine studies. Figure 2 shows the distribution of the studies across the regions.

### 3.3. Pool Prevalence of DENV in Ghana

**Figure 3:**
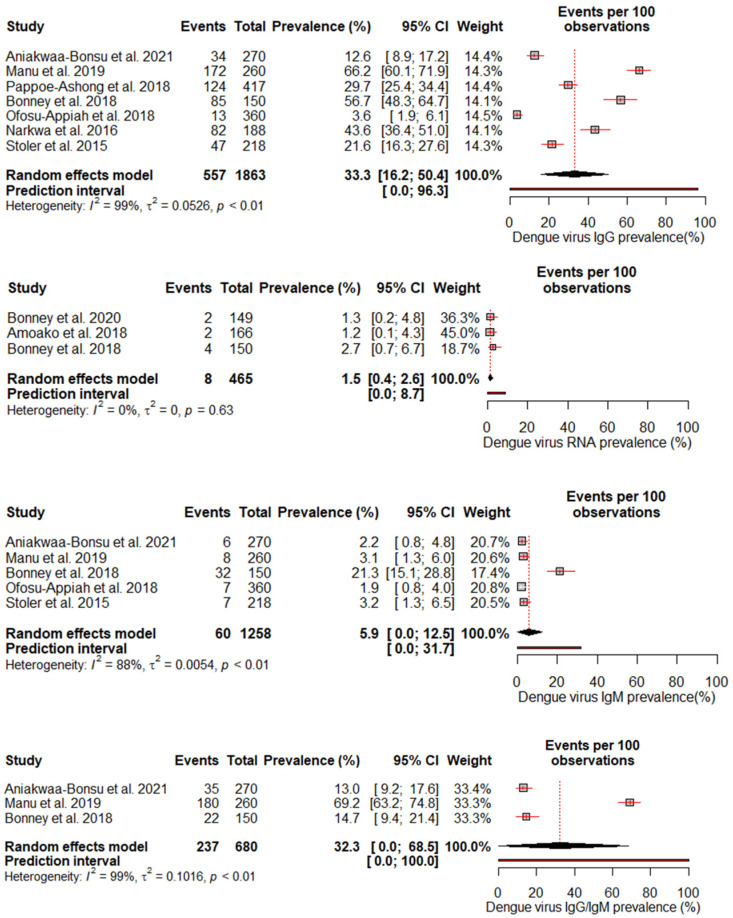
The pooled seroprevalence estimates for different DENV antibody markers (IgG, IgM, RNA, and IgG/IgM combined) reported in the included studies [18,19,23,24,25,26,27,28,29]. The forest plot displays the individual study estimates, the pooled seroprevalence with 95% confidence intervals, and the heterogeneity statistics (I^2^).

A random effects meta-analysis evaluated the pooled prevalence of DENV infection in Ghana. For IgM, the analysis estimated a pooled prevalence of 5.9% (95% CI: 0–12.5%), with high heterogeneity (I^2^ = 88%, *p* < 0.01), indicating relatively low recent infection rates at the population level. The pooled IgG incidence was greater at 33.3% (95% CI: 16.2–50.4%), again with significant heterogeneity (I^2^ = 99%, *p* < 0.01), revealing extensive past exposures in Ghana. The detection of DENV RNA by PCR revealed a pooled prevalence of 1.5% (95% CI: 0.4–2.6%), with no heterogeneity, which is consistent with the low current viremic phase. Studies reporting combined IgG/IgM prevalence estimated a pooled 32.3% (95% CI: 0–68.5%), with substantial heterogeneity (I^2^ = 99%, *p* < 0.01) (Figure 3).

### 3.4. Risk of Bias

The quality of the included studies was assessed via the Joanna Briggs Institute (JBI) critical appraisal tool. The nine-item risk of bias checklist scores are presented in Table S2.

Most studies (*n* = 6) [19,23,24,25,27,29] achieved a ‘100%’ rating, indicating a low risk of bias, as they adequately reported sample frames, sampling methods, sample sizes, settings/subjects, data analysis coverage, diagnostic validity, condition measurement, and statistical analysis. Two studies with scores of ‘88.9%’ were considered to have a high risk of bias because of inadequate sampling and setting descriptions [18,26]. One study scored ‘88.9%’ with an unclear risk of bias because of a lack of detail on the statistical methods used [28]. Overall, the included studies were high quality, with clear reporting of methodological domains, enhancing confidence in their findings on DENV infection patterns in Ghana.

## 4. Discussion

This meta-analysis provides valuable insights into the epidemiology of DENV infection in Ghana. The estimated overall IgG seroprevalence was 33.3%, indicating widespread exposure to DENV in the population. However, there was significant heterogeneity (I^2^ = 99%), indicating that the prevalence differed depending on location, time period, or other study characteristics. This underscores the need for further standardized surveillance across diverse regions. Our findings surpass those of other meta-analyses. For example, our IgG seroprevalence is higher than the 21% reported in Ethiopia [30], the 15.6% reported in a 2019 meta-analysis in Africa [31], and the 14% documented in a 2021 meta-analysis in Africa [32]. Additionally, it exceeds the 25% reported in sub-Saharan Africa [33], though it is lower than the 38.3% found in a meta-analysis conducted in India [34].

We found a pooled IgM prevalence of only 5.9% and 1.5% DENV RNA detection, suggesting that current infection rates may be relatively low. The relatively low pooled estimate suggests that a small proportion of the population has experienced recent/acute infection at any given time. While RNA confirmation of acute cases is more standardized, low-test numbers may underestimate true burdens. However, the high heterogeneity (I^2^ = 88%) for IgM prevalence indicates considerable variability between studies, which could be due to factors such as seasonality of outbreaks or regional differences in transmission intensity. Interestingly, no heterogeneity was observed for the RNA prevalence despite the limited amount of data. This suggests that molecular diagnostic methods may provide more consistent estimates than antibody assays, which can be subject to interassay variability [35]. Our findings suggest that molecular diagnosis could offer a relatively consistent approach when optimized diagnostic algorithms are employed nationally. Notably, the meta-analysis by Simo et al. [31] revealed a lower IgM prevalence of 3.5%, and no DENV RNA detection was identified in Africa. However, the meta-analysis by Eltom et al. [33] reported a higher IgM prevalence and DENV RNA prevalence of 10% and 14%, respectively, in sub-Saharan Africa; similarly, the meta-analysis in Ethiopia revealed a higher IgM prevalence of 9% and a pooled DENV-RNA prevalence of 48% [30].

The identification of DENV-2 and DENV-3 as the predominant serotypes circulating in Ghana provides some important insights but also leaves many unanswered questions. DENV-2 was consistently identified across multiple studies from 2011 to 2018, indicating that it is well-established locally with ongoing endemic transmission. However, without genetic sequencing data, it is difficult to determine whether this represents a stable lineage or repeated reintroductions. The presence of DENV-3 confirms that it also maintains local transmission chains, although, again, the phylogenetic context is lacking. An intriguing finding is that DENV-1 and DENV-4 were not detected, even though they are common in West Africa. Evidence suggests that DENV-1 was introduced from Southern Asia and DENV-4 from Southeast Asia, both establishing themselves in the region over time [36]. However, a recent review indicated that DENV-2 is now more prevalent in West Africa compared to DENV-1 and DENV-4 [37]. The circulation of DENV-2 and DENV-3 in Ghana is worrying since these serotypes can cause a spectrum of disease severity. For example, DENV-2 and DENV-3 are often associated with more severe disease manifestations, ranging from mild dengue fever to severe dengue hemorrhagic fever (DHF) and dengue shock syndrome (DSS), compared to DENV-1 and DENV-4 [38]. This difference can be linked to the structural variations in proteins, such as the envelope (E) protein, which influences virus–host cell interactions and immune response [39]; the membrane (M) protein, which is involved in the immune evasion mechanisms of the pathogen [40,41]; and the capsid (C) protein, which has been shown to contribute to the virulence of the pathogen [38].

Similarly, the case–fatality ratio (CFR) of various DENV serotypes can differ significantly. For instance, studies have shown that DENV-2 has been associated with higher CFRs during outbreaks [42,43]. This highlights the public health implications and the need for targeted interventions for specific serotypes. When a person who has been infected with one DENV serotype is later infected with a different serotype, the non-neutralizing antibodies from the first infection can enhance the severity of the second infection. This process, known as Antibody-Dependent Enhancement (ADE), can lead to more severe disease outcomes [44]. The risk of ADE poses a challenge for dengue vaccine development, as vaccines need to provide balanced immunity against all four serotypes to avoid the potential for vaccine-induced ADE [45]. This should be taken into account when developing therapeutic strategies and public health policies. For instance, Dengvaxia, a live attenuated tetravalent vaccine, provides immunity against all four DENV serotypes [46]. It is recommended for individuals with a previous dengue infection, but its efficacy varies among serotypes (higher against DENV-3 and DENV-4) and is influenced by age and serostatus, making universal vaccination challenging.

While DENV-2 and DENV-3 cause a substantial burden globally, their propensity to cause severe dengue versus milder outcomes varies substantially by region due to factors such as population-level immunity and socioeconomics [32,47]. Vietnam studies reported significant urban–rural and regional differences, with prevalence gradients decreasing from densely populated south-central localities towards forested highlands, corroborating landscape influences on vector dynamics [48,49]. Similarly, a study in Brazil reported higher exposure burdens in Amazonian municipalities than in non-Amazonian municipalities, which was attributed to ecological factors affecting mosquito proliferation [50]. To obtain a clearer picture of serotype dynamics in Ghana, expanded sentinel surveillance with annual whole-genome sequencing is needed from multiple urban and rural sites. This could track whether strains are persistent over decades or periodically displaced by new introductions, with implications for developing adaptive control programs. Further serotyping of symptomatic versus asymptomatic infections may also provide clues about changes in virulence.

RT–PCR, ELISA, and combination methods are established approaches for DENV detection. RT–PCR enables sensitive molecular confirmation of acute infection, but its availability is often limited to well-resourced laboratories. ELISA serology provides broader exposure assessment over a lifetime, but reliability depends heavily on the antigen/antibody kits employed between settings [51]. Inconsistencies in standardizing methods, cut-offs and timing of specimen collection complicated efforts to meaningfully aggregate findings. Moving forward, national guidelines standardizing the serial use of both modalities according to accepted algorithms will optimize disease-phase resolution, critical for guiding clinical management and surveillance goals.

### Limitations and Recommendations

The studies included in our review targeted a range of population cohorts to gain insights into DENV infection in Ghana. As the majority of the included studies were hospital-based and involved acute fever patients, the prevalence estimates may overestimate the “true” community burden. Hospital-based studies provide a useful starting point for detecting clinical cases, but their populations are biased toward individuals able and willing to seek healthcare, precluding measuring the true prevalence across communities, particularly for mild/asymptomatic cases. The inclusion of apparently healthy blood donors offered an alternative perspective, but the generalizability of these convenience samples to the whole country is questionable. It is important to note that the high level of heterogeneity observed in the pooled seroprevalence estimates is a significant limiting factor of this review. The substantial heterogeneity (I^2^ = 99%) in the IgG seroprevalence data suggests that the included studies varied considerably in their findings, which may be associated with differences in the study populations, diagnostic methods, sample types, and other contributing factors. In the future, well-designed population-level serosurveys sampling all age groups are needed for more accurate risk profiling. This allows us to stratify analyses by demographics and local risk factors.

## 5. Conclusions

This review establishes the prevalence of DENV and the circulation of various serogroups in Ghana. The increased seropositivity and heterogeneity underscore the need for standardized, large-scale surveillance to optimize the characterization of the disease and guide appropriate control measures. Integrating dengue prevention strategies into existing vector control programs in Ghana could help reduce the risks posed by this disease.

## Data Availability

All the supporting data are presented in the manuscript and Supplementary Files.

## Supplementary Materials

The following supporting information can be downloaded at: https://www.mdpi.com/article/10.3390/diseases13040114/s1, Figure S1: Funnel plot of dengue virus IgM prevalence studies; Figure S2: Funnel plot of dengue virus IgG prevalence studies; Figure S3: Funnel plot of dengue virus RNA prevalence studies; Figure S4: Funnel plot of dengue virus IgG/IgM prevalence studies; Table S1: Characteristics of included studies; Table S2: JBI’s critical appraisal of studies.

## References

[1] Sabir M.J., Al-Saud N.B.S., Hassan S.M. (2021). Dengue and human health: A global scenario of its occurrence, diagnosis and therapeutics. Saudi J. Biol. Sci..

[2] Murugesan A., Manoharan M. (2020). Dengue Virus. Emerging and Reemerging Viral Pathogens.

[3] Diamond M.S., Pierson T.C. (2015). Molecular Insight into Dengue Virus Pathogenesis and Its Implications for Disease Control. Cell.

[4] Sinha S., Singh K., Ravi Kumar Y.S., Roy R., Phadnis S., Meena V., Bhattacharyya S., Verma B. (2024). Dengue virus pathogenesis and host molecular machineries. J. Biomed. Sci..

[5] Dhole P., Zaidi A., Nariya H.K., Sinha S., Jinesh S., Srivastava S. (2024). Host Immune Response to Dengue Virus Infection: Friend or Foe?. Immuno.

[6] Kularatne S.A., Dalugama C. (2022). Dengue infection: Global importance, immunopathology and management. Clin. Med..

[7] Dengue and Severe Dengue. https://www.who.int/news-room/fact-sheets/detail/dengue-and-severe-dengue.

[8] Singh R.K., Tiwari A., Satone P.D., Priya T., Meshram R.J. (2023). Updates in the management of dengue shock syndrome: A comprehensive review. Cureus.

[9] Uzair H., Waseem R., Kumar N., Hussain M.S., Shah H.H. (2023). Fatal outcome of dengue fever with multi-organ failure and hemorrhage: A case report. SAGE Open Med. Case Rep..

[10] Ilic I., Ilic M. (2024). Global Patterns of Trends in Incidence and Mortality of Dengue, 1990–2019: An Analysis Based on the Global Burden of Disease Study. Medicina.

[11] Schaefer T.J., Panda P.K., Wolford R.W. (2024). Dengue Fever. BMJ Best Pract..

[12] Nakase T., Giovanetti M., Obolski U., Lourenço J. (2024). Population at risk of dengue virus transmission has increased due to coupled climate factors and population growth. Commun. Earth Environ..

[13] Dengue—Global Situation. https://www.who.int/emergencies/disease-outbreak-news/item/2024-DON518.

[14] Lessa C.L., Hodel K.V., Gonçalves M.D., Machado B.A. (2023). Dengue as a disease threatening global health: A narrative review focusing on Latin America and Brazil. Trop. Med. Infect. Dis..

[15] Hossain M.S., Noman A.A., Mamun S.M., Mosabbir A.A. (2023). Twenty-two years of dengue outbreaks in Bangladesh: Epidemiology, clinical spectrum, serotypes, and future disease risks. Trop. Med. Health.

[16] Gainor E.M., Harris E., LaBeaud A.D. (2022). Uncovering the Burden of Dengue in Africa: Considerations on Magnitude, Misdiagnosis, and Ancestry. Viruses.

[17] HEALTH ALERT ON DENGUE FEVER—Ministry Of Health [Internet]. https://www.moh.gov.gh/health-alert-on-dengue-fever/.

[18] Stoler J., Delimini R.K., Bonney J.K., Oduro A.R., Owusu-Agyei S., Fobil J.N., Awandare G.A. (2015). Evidence of recent dengue exposure among malaria parasite-positive children in three urban centers in Ghana. Am. J. Trop. Med. Hygiene..

[19] Narkwa P.W., Mutocheluh M., Kwofie T.B., Owusu M., Annan A., Ali I., Boamah J.K. (2016). Dengue virus exposure among blood donors in Ghana. J. Med. Biomed. Sci..

[20] Page M.J., McKenzie J.E., Bossuyt P.M., Boutron I., Hoffmann T.C., Mulrow C.D., Shamseer L., Tetzlaff J.M., Akl E.A., Brennan S.E. (2020). The PRISMA 2020 statement: An updated guideline for reporting systematic reviews. BMJ.

[21] Ouzzani M., Hammady H., Fedorowicz Z., Elmagarmid A. (2016). Rayyan—A web and mobile app for systematic reviews. Syst. Rev..

[22] JBI Critical Appraisal Tools|JBI. https://jbi.global/critical-appraisal-tools.

[23] Aniakwaa-Bonsu E., Amoako-Sakyi D., Dankwa K., Prah J.K., Nuvor S.V. (2021). Seroprevalence of Dengue Viral Infection among Adults Attending the University of Cape Coast Hospital. Adv. Infect. Dis..

[24] Amoako N., Duodu S., Dennis F.E., Bonney J.H., Asante K.P., Ameh J., Mosi L., Hayashi T., Agbosu E.E., Pratt D. (2018). Detection of dengue virus among children with suspected malaria, Accra, Ghana. Emerg. Infect. Dis..

[25] Bonney J.H., Asigbee T.W., Kotey E., Attiku K., Asiedu-Bekoe F., Mawuli G., Bonney E.Y., Asante I.A., Abana C., Pratt D. (2020). Molecular detection of viral pathogens from suspected viral hemorrhagic fever patients in Ghana. Health Sci. Investig. J..

[26] Bonney J.H., Hayashi T., Dadzie S., Agbosu E., Pratt D., Nyarko S., Asiedu-Bekoe F., Ido E., Sarkodie B., Ohta N. (2018). Molecular detection of dengue virus in patients suspected of Ebola virus disease in Ghana. PLoS ONE.

[27] Manu S.K., Bonney J.H., Pratt D., Abdulai F.N., Agbosu E.E., Frimpong P.O., Adiku T.K. (2019). Arbovirus circulation among febrile patients at the greater Accra Regional Hospital, Ghana. BMC Res. Notes.

[28] Pappoe-Ashong P.J., Ofosu-Appiah L.H., Mingle J.A., Jassoy C. (2018). Seroprevalence of dengue virus infections in Ghana. East Afr. Med. J..

[29] Ofosu-Appiah L., Kutame R., Ayensu B., Bonney J., Boateng G., Adade R., Opare D., Odoom J. (2018). Detection of Dengue Virus in Samples from Suspected Yellow Fever Cases in Ghana. Microbiol. Res. J. Int..

[30] Nigussie E., Atlaw D., Negash G., Gezahegn H., Baressa G., Tasew A., Zembaba D. (2024). A dengue virus infection in Ethiopia: A systematic review and meta-analysis. BMC Infect Dis..

[31] Simo F.B., Bigna J.J., Kenmoe S., Ndangang M.S., Temfack E., Moundipa P.F., Demanou M. (2019). Dengue virus infection in people residing in Africa: A systematic review and meta-analysis of prevalence studies. Sci. Rep..

[32] Mwanyika G.O., Mboera L.E., Rugarabamu S., Ngingo B., Sindato C., Lutwama J.J., Paweska J.T., Misinzo G. (2021). Dengue Virus Infection and Associated Risk Factors in Africa: A Systematic Review and Meta-Analysis. Viruses.

[33] Eltom K., Enan K., El Hussein A.R.M., Elkhidir I.M. (2021). Dengue Virus Infection in Sub-Saharan Africa Between 2010 and 2020: A Systematic Review and Meta-Analysis. Front. Cell. Infect. Microbiol..

[34] Ganeshkumar P., Murhekar M.V., Poornima V., Saravanakumar V., Sukumaran K., Anandaselvasankar A., John D., Mehendale S.M. (2018). Dengue infection in India: A systematic review and meta-analysis. PLoS Negl. Trop. Dis..

[35] Raafat N., Blacksell S.D., Maude R.J. (2019). A review of dengue diagnostics and implications for surveillance and control. Trans. R. Soc. Trop. Med. Hyg..

[36] Alfsnes K., Eldholm V., Gaunt M.W., de Lamballerie X., Gould E.A., Pettersson J.H.O. (2021). Tracing and tracking the emergence, epidemiology and dispersal of dengue virus to Africa during the 20th century. One Health.

[37] Mercy K., Youm E., Aliddeki D., Faria N.R., Kebede Y., Ndembi N. (2024). The Looming Threat of Dengue Fever: The Africa Context. Open Forum Infect Dis..

[38] Yung C.F., Lee K.S., Thein T.L., Tan L.K., Gan V.C., Wong J.G., Lye D.C., Ng L.C., Leo Y.S. (2015). Dengue Serotype-Specific Differences in Clinical Manifestation, Laboratory Parameters and Risk of Severe Disease in Adults, Singapore. Am. Soc. Trop. Med. Hyg..

[39] Fibriansah G., Lim X.N., Lok S.M. (2021). Morphological Diversity and Dynamics of Dengue Virus Affecting Antigenicity. Viruses.

[40] Goh G.K.M., Dunker A.K., Uversky V.N. (2016). Correlating Flavivirus virulence and levels of intrinsic disorder in shell proteins: Protective roles vs. immune evasion. Mol. Biosyst..

[41] Goh G., Dunker A., Foster J., Uversky V. (2019). Zika and Flavivirus Shell Disorder: Virulence and Fetal Morbidity. Biomolecules.

[42] Dumre S.P., Bhandari R., Shakya G., Shrestha S.K., Cherif M.S., Ghimire P., Klungthong C., Yoon I.K., Hirayama K., Na-Bangchang K. (2017). Dengue Virus Serotypes 1 and 2 Responsible for Major Dengue Outbreaks in Nepal: Clinical, Laboratory, and Epidemiological Features. Am. J. Trop. Med. Hyg..

[43] Vicente C.R., Herbinger K.H., Fröschl G., Malta Romano C., de Souza Areias Cabidelle A., Cerutti Junior C. (2016). Serotype influences on dengue severity: A cross-sectional study on 485 confirmed dengue cases in Vitória, Brazil. BMC Infect. Dis..

[44] Guzman M.G., Vazquez S. (2010). The Complexity of Antibody-Dependent Enhancement of Dengue Virus Infection. Viruses.

[45] Aynekulu Mersha D.G., van der Sterren I., van Leeuwen L.P., Langerak T., Hakim M.S., Martina B., van Lelyveld S.F., van Gorp E.C. (2024). The role of antibody-dependent enhancement in dengue vaccination. Trop. Dis. Travel. Med. Vaccines.

[46] Nivarthi U.K., Swanstrom J., Delacruz M.J., Patel B., Durbin A.P., Whitehead S.S., Kirkpatrick B.D., Pierce K.K., Diehl S.A., Katzelnick L. (2021). A tetravalent live attenuated dengue virus vaccine stimulates balanced immunity to multiple serotypes in humans. Nat. Commun..

[47] Garcia-Bates T.M., Cordeiro M.T., Nascimento E.J., Smith A.P., Soares de Melo K.M., McBurney S.P., Evans J.D., Marques E.T., Barratt-Boyes S.M. (2013). Association between Magnitude of the Virus-Specific Plasmablast Response and Disease Severity in Dengue Patients. J. Immunol..

[48] Huynh T.T.T., Minakawa N. (2022). A comparative study of dengue virus vectors in major parks and adjacent residential areas in Ho Chi Minh City, Vietnam. PLoS Negl. Trop. Dis..

[49] Pham H.V., Doan H.T., Phan T.T., Tran Minh N.N. (2011). Ecological factors associated with dengue fever in a central highlands Province, Vietnam. BMC Infect. Dis..

[50] Lowe R., Lee S., Martins Lana R., Torres Codeço C., Castro M.C., Pascual M. (2020). Emerging arboviruses in the urbanized Amazon rainforest. BMJ.

[51] Kabir M.A., Zilouchian H., Younas M.A., Asghar W. (2021). Dengue Detection: Advances in Diagnostic Tools from Conventional Technology to Point of Care. Biosensors.

