# Supplementary material for: Seroprevalence and Serotypes of Dengue Virus Infection in Ghana: A Systematic Review and Meta-Analysis
Source: Diseases. 2025 Apr 14;13(4):114. doi: 10.3390/diseases13040114 (PMC12025918; doi:10.3390/diseases13040114)
Supplement: Supplementary file 1 [file diseases-13-00114-s001.zip › diseases-3260602-supplementary.pdf]

Table S1. Characteristics of included studies

Study	Study design	Study settings	Region	Study period	Sample size	Types of samples	Target population	Time frame	Type of diagnostic technique	Type of detected biological markers	IgG	IgM	RNA	IgG/IgM	Serotype
Aniakwaa-Bonsu et al. 2021	Cross-sectional	Clinical	Central	Feb-Jul 2019	270	Blood	Adult 18 and above at least presenting with fever and three malaria-like symptoms	Out of outbreak	RDT ELISA	IgG IgM	34	6	-	35	-
Amoako et al. 2018	Cross-sectional	Clinical	Brong Ahafo, Greater Accra	Oct 2016-Jul 2017	166	Blood	Children 1-15 years of age with fever and suspected of malaria	Out of outbreak	RT-PCR	RNA	-	-	2	-	DENV-2
Bonney et al. 2020	Case study	Clinical	Ashanti, Bono Ahafo, Central, Eastern, Western, Volta, Northern, Upper East, Greater Accra	Jan 2017-Dec 2018	149	Blood	Suspected viral hemorrhagic fever patients	Out of outbreak	RT-PCR	RNA	-	-	2	-	DENV-2
Bonney et al. 2018	-	Clinical	Ashanti, Bono Ahafo, Central, Eastern, Western, Volta, Northern, Upper East, Upper West, Greater Accra	2014-2016	150	Blood	Patient suspected of Ebola virus disease	Outbreak	RT-PCR ELISA	IgG IgM RNA	85	32	4	22	DENV-2 DENV-3
Manu et al. 2019	Cross-sectional	Clinical	Greater Accra	May 2016-April 2017	260	Blood	Suspected dengue and/or chikungunya febrile patients	Out of outbreak	RT-PCR ELISA	IgG IgM RNA	172	8	-	180	-
Narkwa et al. 2016	Cross-sectional	Clinical	Brong Ahafo, Ashanti	Feb 2013-Dec 2015	188	Blood	Healthy blood donors	Out of outbreak	RT-PCR ELISA	IgG IgM RNA	82	-	-	-	-
Pappoe-Ashong et al. 2018	Cross-sectional	Clinical	Ashanti, Bono Ahafo, Central, Eastern, Western, Volta, Northern, Upper East, Upper West, Greater Accra	Jan-De 2014	417	Blood	Patient with fever and jaundice	Outbreak	ELISA	IgG IgM	124	-	-	-	-
Stoler et al. 2015	-	Clinical	Brong Ahafo, Greater Accra, Upper East	2011-2014	218	Blood (plasma)	Children ages 2-14 years with confirmed malaria	Out of outbreak	RT-PCR ELISA	IgG IgM RNA	47	7	-	-	-
Ofosu-Appiah et al. 2018	Cross-sectional	Clinical	Ashanti, Bono Ahafo, Central, Eastern, Western, Volta, Northern,	Jan-Dec 2013	360	Blood	Patient aged 6 months to 82 years suspected of yellow fever	Out of outbreak	RT-PCR ELISA	IgG IgM RN A	13	7	-	-	-

			Upper East,<br>Upper West,<br>Greater Accra												

Table S2. JBI's critical appraisal of studies

		JBI's critical appraisal questions												
Author & Year	Total Sample Size	Q1	Q2	Q3	Q4	Q5	Q6	Q7	Q8	Q9	Score	%	Study quality	Overall appraisal
Aniakwaa-Bonsu et al. 2021	270	1	1	1	1	1	1	1	1	1	9	100	High	Included
Amoako et al. 2018	166	1	1	1	1	1	1	1	1	1	9	100	High	Included
Bonney et al. 2020	149	1	1	1	1	1	1	1	1	1	9	100	High	Included
Bonney et al. 2018	150	1	1	1	0	1	1	1	1	1	8	88.9	High	Included
Manu et al. 2019	260	1	1	1	1	1	1	1	1	1	9	100	High	Included
Narkwa et al. 2016	188	1	1	1	1	1	1	1	1	1	9	100	High	Included
Pappoe-Ashong et al. 2018	417	1	1	1	1	1	1	1	0	1	8	88.9	High	Included
Stoler et al. 2015	218	1	1	1	0	1	1	1	1	1	8	88.9	High	Included
Ofosu-Appiah et al. 2018	360	1	1	1	1	1	1	1	1	1	9	100	High	Included

Q1. Was the sample frame appropriate to address the target population?

Q2. Were study participants sampled in an appropriate way?

Q3. Was the sample size adequate?

Q4. Were the study subjects and the setting described in detail?

Q5. Was the data analysis conducted with sufficient coverage of the identified sample?

Q6. Were valid methods used for the identification of the condition?

Q7. Was the condition measured in a standard, reliable way for all participants?

Q8. Was there appropriate statistical analysis?

Q9. Was the response rate adequate, and if not, was the low response rate managed appropriately?

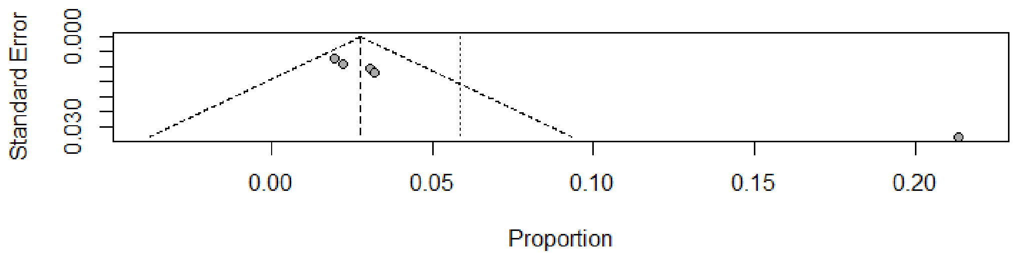

Figure S1. Funnel plot of dengue virus IgM prevalence studies

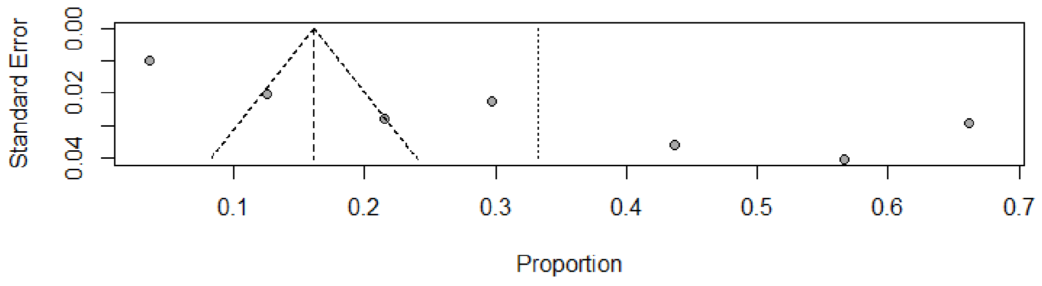

Figure S2. Funnel plot of dengue virus IgG prevalence studies

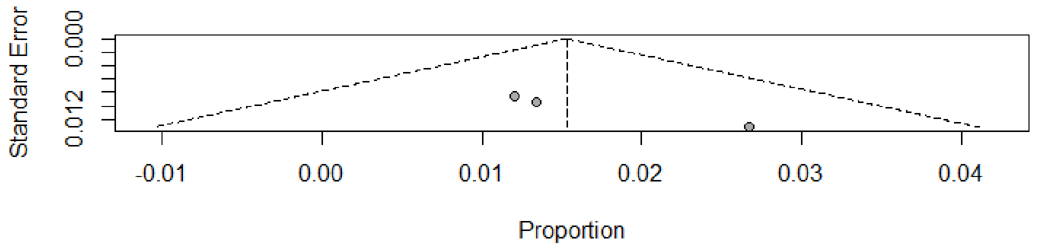

Figure S3. Funnel plot of dengue virus RNA prevalence studies

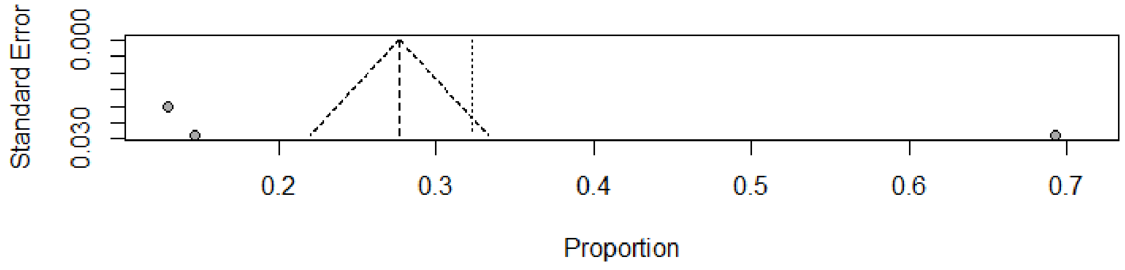

Figure S4. Funnel plot of dengue virus IgG/IgM prevalence studies
